# Quantitative imaging to evaluate malignant potential of IPMNs

**DOI:** 10.18632/oncotarget.11769

**Published:** 2016-08-31

**Authors:** Alexander N. Hanania, Leonidas E. Bantis, Ziding Feng, Huamin Wang, Eric P. Tamm, Matthew H. Katz, Anirban Maitra, Eugene J. Koay

**Affiliations:** ^1^ University of Texas Medical School, Houston, TX, USA; ^2^ Department of Radiation Oncology, University of Texas MD Anderson Cancer Center, Houston, TX, USA; ^3^ Department of Biostatistics, University of Texas MD Anderson Cancer Center, Houston, TX, USA; ^4^ Department of Pathology, University of Texas MD Anderson Cancer Center, Houston, TX, USA; ^5^ Department of Diagnostic Radiology, University of Texas MD Anderson Cancer Center, Houston, TX, USA; ^6^ Department of Surgical Oncology, University of Texas MD Anderson Cancer Center, Houston, TX, USA

**Keywords:** IPMN, quantitative imaging, radiomics, fukuoka, pancreatic cyst

## Abstract

**Objective:**

To investigate using quantitative imaging to assess the malignant potential of intraductal papillary mucinous neoplasms (IPMNs) in the pancreas.

**Background:**

Pancreatic cysts are identified in over 2% of the population and a subset of these, including intraductal papillary mucinous neoplasms (IPMNs), represent pre-malignant lesions. Unfortunately, clinicians cannot accurately predict which of these lesions are likely to progress to pancreatic ductal adenocarcinoma (PDAC).

**Methods:**

We investigated 360 imaging features within the domains of intensity, texture and shape using pancreatic protocol CT images in 53 patients diagnosed with IPMN (34 “high-grade” [HG] and 19 “low-grade” [LG]) who subsequently underwent surgical resection. We evaluated the performance of these features as well as the Fukuoka criteria for pancreatic cyst resection.

**Results:**

In our cohort, the Fukuoka criteria had a false positive rate of 36%. We identified 14 imaging biomarkers within Gray-Level Co-Occurrence Matrix (GLCM) that predicted histopathological grade within cyst contours. The most predictive marker differentiated LG and HG lesions with an area under the curve (AUC) of .82 at a sensitivity of 85% and specificity of 68%. Using a cross-validated design, the best logistic regression yielded an AUC of 0.96 (σ = .05) at a sensitivity of 97% and specificity of 88%. Based on the principal component analysis, HG IPMNs demonstrated a pattern of separation from LG IPMNs.

**Conclusions:**

HG IPMNs appear to have distinct imaging properties. Further validation of these findings may address a major clinical need in this population by identifying those most likely to benefit from surgical resection.

## INTRODUCTION

Pancreatic cysts are identified in greater than 2% of the general patient population who present with symptoms unrelated to the pancreas [1, 2]. The striking increase in the prevalence of asymptomatic pancreatic cysts can be attributed to the increased use of abdominal imaging (CT or MRI); currently, more than 50 million such scans are performed annually in the United States. The prevalence of this “man-made epidemic” is only expected to rise, and it is hard to predict the definitive course of such cysts without surgical resection.

Retrospective histopathological studies on resected pancreatic cysts have shown that approximately half are intraductal papillary mucinous neoplasms (IPMNs) [3–5]. IPMNs are a subset of pancreatic cysts that are often detectable on imaging; however, the inability to accurately predict which cysts are indolent and which either have an infiltrating pancreatic ductal adenocarcinoma (PDAC) or are highly likely to progress to a PDAC remains a clinical challenge. Patients with non-invasive IPMNs have a 5-year survival rate of 90-100% following surgical resection, while those who had an IPMN with an associated invasive component have long-term survival reduced by nearly half [6–9], reiterating the dire importance of early identification of high risk lesions that are likely to progress to pancreatic cancer. While the post-operative mortality rate from pancreatic surgery has significantly decreased in the last two decades, there remains a low but serious risk of major or minor post-operative morbidity [5, 10], underscoring the need to design strategies that would help to avoid over-treating individuals at low risk who can be followed without need of surgery. The current, internationally accepted, consensus-based, Fukuoka criteria is an approach to pre-operatively identifying malignant IPMNs and is often effective; however, it remains imperfect, incorrectly labeling benign lesions for surgery 1/3rd of the time at high volume and experienced centers [11–13].

Quantitative imaging, sometimes referred to as “Radiomics,” has the potential to provide a higher level of care to a large number of patients. This imaging analysis utilizes algorithms to derive image texture [14], shape and physical transport properties [15, 16], as well as bio-statistical tools for clinical prediction models. To address this significant healthcare burden and clinical conundrum, we evaluated the use of quantitative imaging features on preoperative CT scans to determine associations of these features with the degree of dysplasia seen in the resected specimens. Our hypothesis was that high grade and/or invasive IPMNs would have distinct imaging features compared to IPMNs with low grade dysplasia. We restricted this analysis to resected specimens (both branch-duct and main-duct) so that histology could be used as the gold standard comparator against quantitative image analysis.

## RESULTS

We identified 53 cases of IPMN (34 “high-grade” and 19 “low-grade”) for quantitative analysis of the pancreatic cysts and pancreas parenchyma to differentiate high grade from low grade lesions. The median age of study participants was 73 years, (range, 54-89 years). Of the 53 cases, their demographics by gender and race are as follows: 57% male and 43% female; 90% Caucasian, 5% Hispanic and 5% African American.

**Figure 1:**
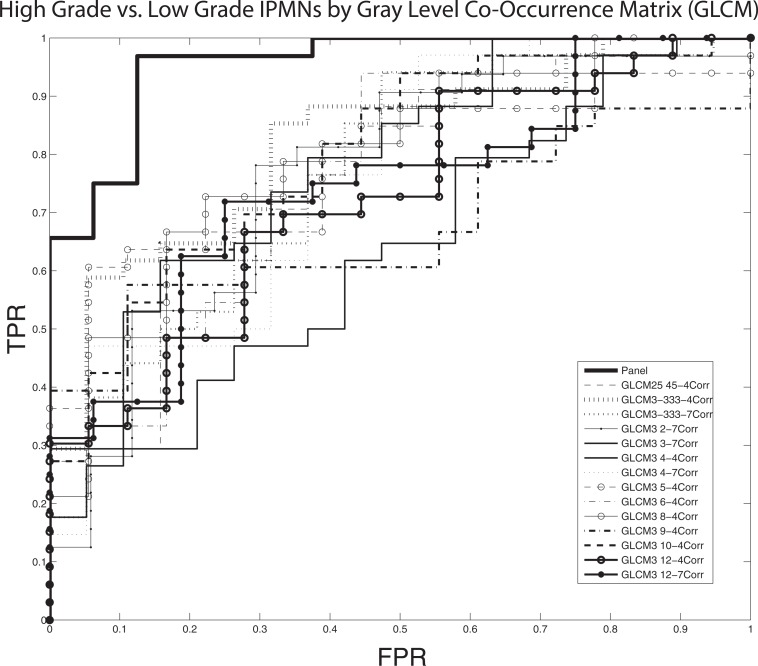
Receiver operating characteristic (ROC) curves demonstrating that pathologically-confirmed, high grade IPMNs are differentiated from low grade IPMNs based on quantitative imaging characteristics generated from a Gray Level Co-Occurrence Matrix (GLCM) A cross-validated, logistic-regression model based on a panel of markers demonstrates an area under the curve (AUC) of 0.96 (σ = .05) at a sensitivity of 97% and specificity of 88% while the best individually performing marker demonstrates AUC of 0.82.

Quantitative imaging analysis of pancreatic cysts yielded 14 top performing markers in terms of area under the curve (AUC) as well as in terms of true positive rate (TPR) at fixed 5% false positive rate (FPR). All markers were within the cyst contour region of interest and within the domain of texture analysis, specifically in Gray-Level Co-Occurrence Matrix (GLCM) using correlation statistics. Receiver operating characteristic curves were constructed for all variables to differentiate between high and low grade IPMNs (Figure 1). The most predictive individual marker resulted in an area under the curve (AUC) of 0.82 (95% CI 0.71-0.93). The maximum of the Youden index was 0.5372 with a corresponding pair of sensitivity and specificity of 85% and 68%, respectively. As an added measure, we conducted a false-discovery rate (FDR) analysis which led to a p-value correction that remained significant for this variable (p = .018).

**Figure 2:**
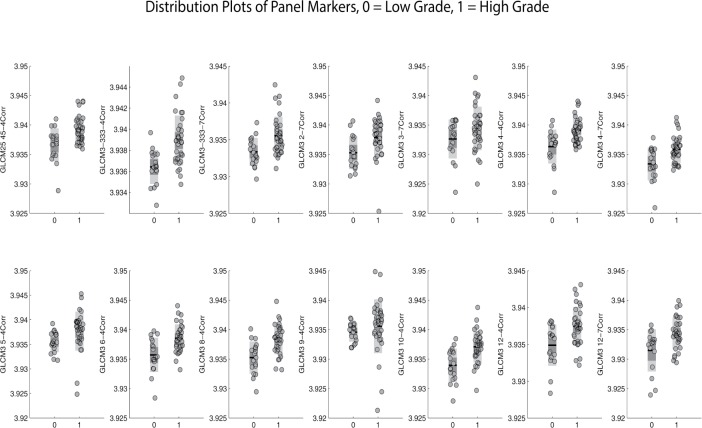
Distribution plots characterizing the performance of individual Gray Level Co-Occurrence Matrix (GLCM) panel markers and their ability to discriminate between high grade and low grade IPMNs

Our cross-validated panel consisted of 10 markers selected from the top 14 candidate markers and had an AUC of 0.96 (95% CI: 0.92-0.99). The associated maximum of the Youden index was 0.8438 and its corresponding pair of sensitivity and specificity was 97% and 88% respectively. (Figures 1, 2, Table 1). The Fukuoka criteria had a false positive rate of 36% in this cohort of resected IPMNs where all cysts were classified as high risk by Fukuoka criteria (Table 2).

**Table 1:** Candidate Markers for Differentiating HG vs. LG IPMNs (*Panel Marker)

Marker	Name	Criteria	AUC	p-value
1	GLCM25 45-4Correlation*	AUC	0.77	7.93E-05
2	GLCM3 -333-4Correlation*	AUC	0.82	2.39E-08
3	GLCM3 -333-7Correlation	AUC	0.77	8.15E-05
4	GLCM3 2-7Correlation*	AUC	0.76	0.000316
5	GLCM3 3-7Correlation	ROC(0.05)	0.64	0.064
6	GLCM3 4-4Correlation	AUC	0.78	1.95E-05
7	GLCM3 4-7Correlation*	AUC	0.76	0.000138
8	GLCM3 5-4Correlation*	BOTH	0.78	1.53E-05
9	GLCM3 6-4Correlation*	AUC	0.76	0.000232
10	GLCM3 8-4Correlation	AUC	0.80	2.22E-06
11	GLCM3 9-4Correlation*	ROC(0.05)	0.68	0.0181
12	GLCM3 10-4Correlation*	AUC	0.80	3.66E-06
13	GLCM3 12-4Correlation*	ROC(0.05)	0.72	0.00299
14	GLCM3 12-7Correlation*	ROC(0.05)	0.74	0.0009

**Table 2:** Worrisome IPMN Characteristics Based on Fukuoka Criteria

IPMN Characteristics		Main Duct	Cyst >3cm	Mural Nodules	MPDD > 5mm	Positive Cytology	Symptomatic Neoplasm
Total (n = 53)		6 (11%)	23 (43%)	7 (13%)	12 (23%)	6 (11%)	10 (19%)
By Grade							
	LG (n = 19)	4 (21%)	10 (53%)	1 (5%)	3 (16%)	2 (11%)	2 (11%)
	HG (n = 34)	2 (6%)	13 (38%)	6 (18%)	9 (26%)	4 (12%)	8 (24%)

MPDD- Main Pancreatic Duct Diameter

**Figure 3:**
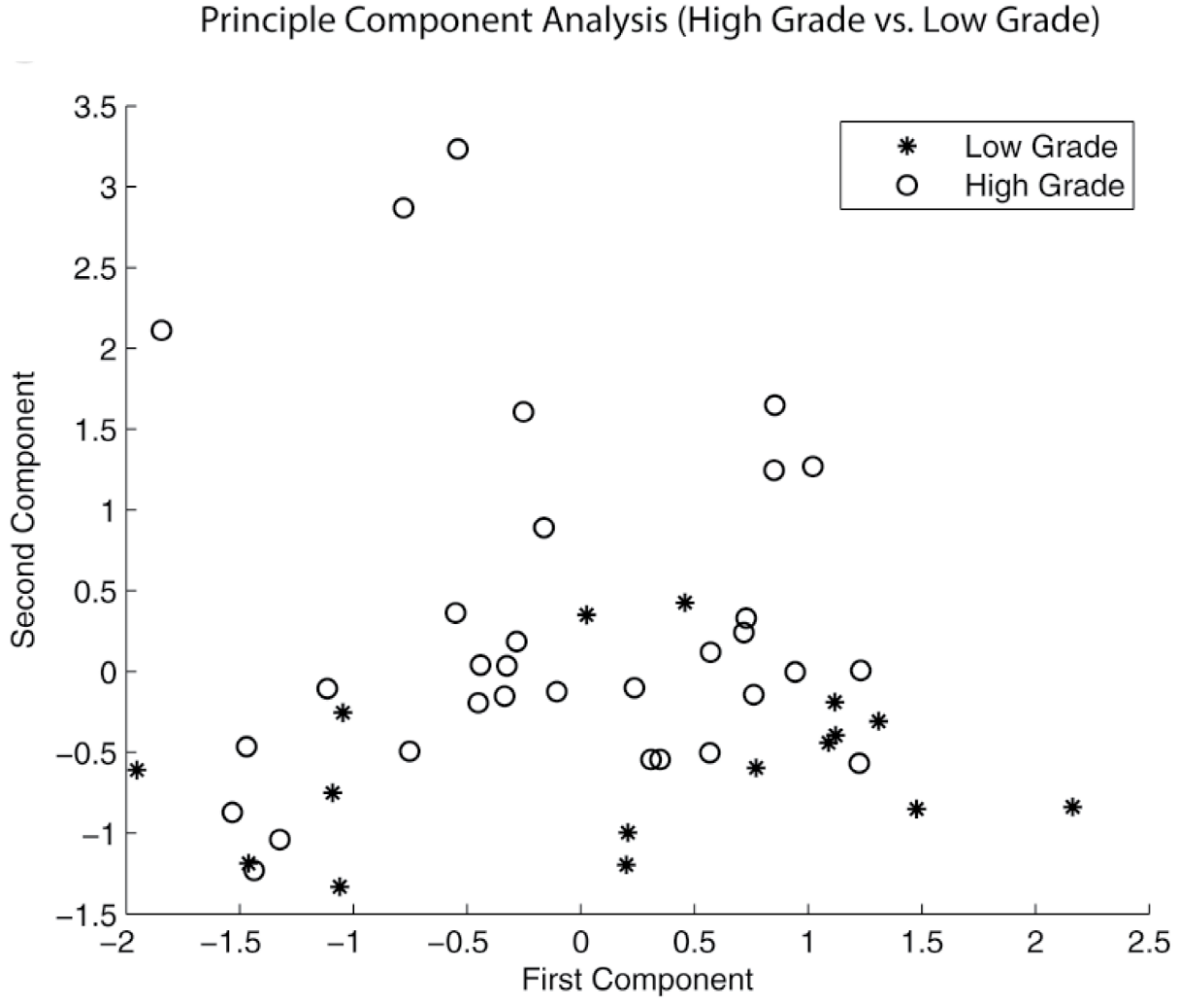
Principle component analysis (PCA) demonstrating a pattern of separation of between high grade and low grade IPMNs This finding was present only in contours of pancreatic cysts and did not exist when PCA was run on pancreas parenchyma.

Finally, in our principle components analysis, the first two components showed that the high grade IPMN scores exhibit a distinct pattern than the corresponding low grade IPMN scores, indicating the quantitative features differentiated these two groups (Figure 3). No such pattern was revealed based on the principal component analysis for the remaining data of the parenchyma and pancreas.

## DISCUSSION

In this retrospective analysis of CT data from patients who underwent resection for IPMN, we were able to correlate histopathological grade of the IPMNs to quantitative features extracted from standard pancreas protocol CT imaging. Multiple statistical analyses consistently demonstrated that quantitative imaging features of IPMNs differentiated high grade/invasive IPMNs from low grade ones.

**Figure 4:**
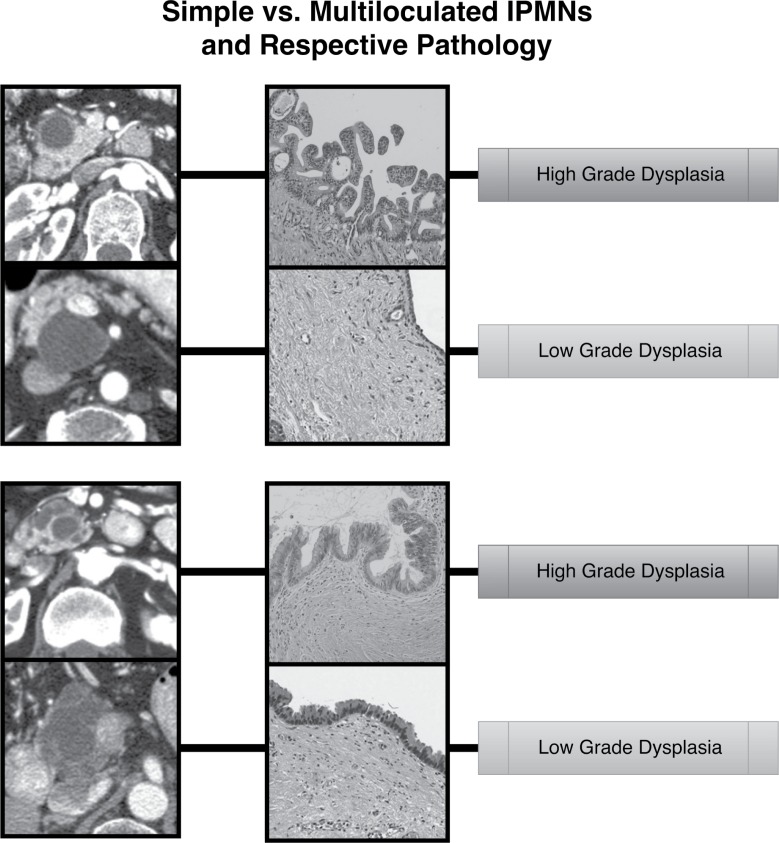
Imaging and respective pathology of several IPMN samples in this study are presented These samples show that cysts that appear grossly simple may harbor high grade dysplasia while suspicious, multi-lobulated cysts may yield only low grade dysplasia. Gross radiologic criteria alone may potentially be improved upon in the future with Radiomic tools that offer greater specificity to discriminate which IPMNs require intervention.

The Fukuoka criteria is a valuable tool in stratifying patients with IPMNs for surgery; however, the high false positive rate in our cohort, which affirms the current literature, further supports the notion that tools with greater specificity are needed to help inform decisions regarding interventions such as surgery or endoscopic ultrasound with fine needle aspiration (EUS-FNA) in patients with worrisome features by Fukuoka criteria. As a case in point regarding the difficulty of classifying IPMNs based on radiologic appearance, we have included four of our samples with imaging and respective histology to show that cysts that appear grossly simple may harbor high grade dysplasia while suspicious, multi-lobulated cysts may yield only low grade dysplasia (Figure 4).

Promising new molecular markers have recently been cited in the literature in a retrospective, multi-institutional paper by Springer and colleagues where they were able to achieve 90-100% sensitivity and 92-98% specificity in classifying pancreatic cysts [17]. Combining these molecular markers with our quantitative imaging features may be used synergistically to provide a robust model to help guide decision making for patients with pancreatic cysts. Further, feature-based imaging approaches similar to this one have also proven fruitful in other solid tumors, such as glioblastoma [18] and lung cancer [19].

Limitations of this study include its retrospective and single institution design as well as small sample size. In addition, as with most imaging biomarker studies, a large number of tested variables may increase the risk of false discovery. We addressed this potential problem by implementing a false discovery analysis, a cross-validated panel, and principal component analysis. Our data also suggest that a higher rate of prediction is possible by constructing a model from multiple variables. We decided to include this panel data without making strong conclusions as this approach may be a consequence of “over-fitting” and could lead to an inaccurate account of validity. Nonetheless, the accuracy we were able to achieve with our cross-validated model warrants further investigation. At present our findings provide a proof of principle and our next step is to evaluate our cross-validated model using data from other institutions, with a particular emphasis on branch duct IPMNs, which present the most challenging cases to manage due to highly variable malignant potential. As we refine our analysis and identify the key features that are associated with malignancy, we will prospectively evaluate these features in patients with IPMN.

Notably, an independent study by Permuth et al [20], conducted at another institution, has also demonstrated the potential of predicting the malignant potential of IPMNs. They identified textural features such as we did (GLCM) as well as non-textural features and miRNA expression levels. Given the nature of radiomic data and the current difficulty of comparing features of computed tomography among differing institutions (varying imaging protocols as well as hardware [CT scanners]), externally validating a model from one institution to make predictions at another remains a challenging task that requires considerable problem-solving; however, we believe that together our independent internally validated discoveries on predictive textural analysis for IPMNs provide a strong argument for future consideration of our findings and cross collaborations.

In conclusion, our findings demonstrate that imaging-based, physical properties of IPMNs associate with their malignant potential and suggest the possibility of a non-invasive method to appropriately select patients for surgical or non-surgical management. In clinical practice, we envision a quantitative tool that provides a measure of malignancy risk for a given IPMN lesion and guides borderline cases to either resection or observation. This decision-making may be combined with clinical criteria and molecular markers to provide a higher degree of certainty. Future implementation of these findings into robust statistical models may provide a more scientifically meaningful and clinically relevant method to characterize IPMNs and fulfill a critical need for this patient population given the associated morbidity, potential mortality and associated health care costs of undergoing surgical intervention.

## MATERIALS AND METHODS

We retrospectively examined patients with IPMN seen at our institution between March 2003 and October 2011 who received a pancreas protocol CT scan, subsequent surgical resection, and pathological diagnosis confirming IPMN. Cases were selected in reverse chronological order based on IPMN specimens that physically exist at our institution. Each specimen was then clinically correlated with patient record number, a procedure note outlining the surgical resection of the IPMN and a pre-operative pancreas protocol CT scan. We did not select patients for inclusion based on the radiographic appearance. All consecutive resected specimens with appropriate, matched imaging were used. We recorded the histological grade of the IPMN from surgical pathology using standard histological criteria [5]. IPMNs with concomitant pancreatic ductal adenocarcinoma (PDAC) or high grade dysplasia (HGD) were lumped together into “high-grade (HG)” while low grade or intermediate dysplasia was assessed as “low-grade (LG).” The 2012 IPMN consensus guidelines were not employed in the period of surgical resection in this study; however, we reviewed cases for features described in the most up-to-date consensus-based Fukuoka criteria (cyst size > 3 cm, mural nodules, main pancreatic duct diameter > 5 mm, positive cytology and symptoms related to the neoplasm) to better characterize our cohort [11]. All cases used in this study were positive by Fukuoka criteria. We then performed detailed analyses of pre-surgical pancreas protocol CT scans obtained in these patients. The study was approved by the Institutional Review Board at MD Anderson Cancer Center, Houston, TX.

We used a process of segmentation, where we contoured several sets that consisted of either: the cyst alone, the cyst and pancreas together, the pancreas excluding the cyst or pancreatic parenchyma. In situations where multiple cysts existed, we contoured the most radiologically suspicious cyst with review by an expert radiologist (ET). For main-branch duct IPMNs where a clear cystic lesion was not clearly identifiable; we contoured the area of the main pancreatic duct with greatest suspicion of harboring the IPMN. In situations where there were associated side branch cysts that made connections to the main branch, the side branch cysts were included in the contour. In a few cases where there was frank invasion of neoplasm into neighboring tissue, our contouring goal was to sample as much of the cystic component as possible; however, solid mural components as well as potential non-cystic components originating from the IPMN were also contoured. For patients with stents or other metallic artifacts, our contours excluded the margin around the artifact to prevent distortion of true signal. We used Velocity software (Varian Medical Systems, Palto Alto, CA) to register portal to arterial phases and to aid in contouring of pancreatic cysts, pancreas and parenchyma. Contours were reviewed independently by a radiation oncologist and radiologist for accuracy and consistency.

A total of 360 physical features were extracted from the arterial phases using IBEX (Imaging Biomarker Explorer) [14], which runs imaging analysis for hundreds of algorithmic-based features. Pre-processing is also a function of the IBEX software and was implemented in our analysis to remove noise from raw images along with edge detection (a method of identifying where brightness discontinues or varies significantly between points). We used IBEX for quantitative analysis of the arterial phase of the CT, to extract physical features of the cysts and the surrounding parenchyma, as we have previously described [15, 16]. We also quantified the degree of heterogeneity within the normal pancreas parenchyma and within the cyst(s) (e.g., mean, standard deviation, entropy, contrast, and kurtosis of the 1D and 2D gray-level intensity distribution) within the segmented area, among many other features that can be extracted and quantified. Venous-phases were collected and likely may provide additional information in future analysis; however, they were not utilized in this study.

Ultimately, the markers we identify are within the domain of Gray-Level Co-Occurrence Matrix (GLCM), a form of texture analysis, which provides statistical measures of the spatial relationship of pixels within an image. In simple terms, GLCM can be understood as a mathematical method to analyze texture. Within GLCM, identified markers in this study were within the correlation statistic which provides a joint probability occurrence of specific pixel pairs. Put simply, we propose that the correlation aspect of GLCM as it relates to our analysis likely provides information regarding physical properties of tissue, specifically the heterogeneity within the neoplasm as well as the tumor-stromal interface.

### Data analysis

In this pilot study, we implemented a trifold approach to our data analysis.

Our primary goal was to identify the top performing markers in the prediction of pathological grade of IPMNs among all 360 imaging variables using receiver operating characteristic (ROC) analysis as well as false-discovery rate (FDR) analysis to control for Type I errors [21]. From these variables we selected the variable with best area under the curve (AUC) as well as the most significant FDR corrected p-value. We used ROC curves to help quantify predictive ability of markers for individual performance and for selection into a synergistic panel. FDR analysis adjusts significance calculations based on number of multiple comparisons, in this case, the number of variables being analyzed and provides adjusted significance. In this manner we attempt to decrease the likelihood of identifying a falsely significant result.

As a secondary goal for this pilot study, we constructed a panel based on the AIC (Akaike Information Criterion) to evaluate whether the use of a model combining a panel of markers could be used to increase diagnostic accuracy. We scrutinized all possible combinations of the top performing markers focusing on markers in terms of AUC as well as in terms of true positive rate (TPR) at fixed 5% false positive rate (FPR), in order to pick the best AIC performing logistic regression model.

For the construction of a panel, we considered as an initial set the top 10 markers in terms of AUC and the top 5 in terms of sensitivity at a FPR = 0.05 (or ROC(0.05)). We obtain a panel after scrutinizing all possible models that can be built by this initial set using AIC as the selection criterion. This panel consists of 10 markers and was cross validated by the following two schemes of leave 1/3-out cross-validation:

(1) Splitting the data into a training set (2/3 of the data) and a test set (1/3) of the data. Fit the model using the training set and apply it to the test obtaining also its AUC. Repeat 1000 times and average out the obtained AUCs. Under this scheme the model is refitted for each training set even though the markers that participate remain the same.

(2) Cross-validate the whole procedure, namely use (2/3) of the data of all 360 markers, select the top 10 in terms of AUC and top 5 in terms of ROC(0.05) in the training set, scrutinize all possible models, build the model (still in the training set), fix its coefficients, and apply it on the test set (10 repetitions).

Regarding the inference for the individual markers as well as for the panel, we used the bootstrap (1000 draws). We note that the variance of the estimated coefficients of the panel was taken into account during the bootstrap by refitting the model for every bootstrap iteration.

Cross-validation provides a method of internally validating our data by minimizing the potential of over-fitting (identifying random noise rather than true signal) by creating a model based on variations of training and testing sets.

Finally, for completeness and to confirm that signal exists within the cysts contours, we implemented a principal component analysis (PCA), which was applied to all markers in all contours (cyst, pancreas, pancreas and cyst, pancreas parenchyma). Using the first two components we are allowed to visualize any potential regarding the discriminatory ability of our markers. PCA is a dimension reduction technique that employs an orthogonal transformation and reduces a set of variables to linearly uncorrelated principal components. The derived principal components account for the most part of the variability of the data. In our study, we collected a large number of variables within a single population, and PCA is a method of affirming that patterns exist among our data.

We also obtained the maximum of the Youden index estimate [22] along with its associated pair of sensitivity and specificity for the best performing individual marker, as well as for the panel. The Youden index (also known as Youden's J statistic) is a statistic which helps us evaluate the potential effectiveness of diagnostic tests. It is defined for all points of the ROC curve, and the maximum value of that index can be considered as an overall summary measure of the performance of a marker. The maximum of the Youden index is obtained by maximizing both the sensitivity and specificity for a given ROC curve. We used that measure to help understand the clinical predictive value of an individual marker and our panel.

MATLAB 2014b (The MathWorks, Inc., Natick, MA) and JMP11 (SAS Institute Inc, Cary, NC) were used for statistical analysis.
